# Percentage of Gutta-Percha-Filled Areas in Canals Obturated by Two Different Core Techniques with Endodontic Bioceramics Sealer

**DOI:** 10.3390/ma19010037

**Published:** 2025-12-21

**Authors:** Antonio Libonati, Danilo Marroni, Giulio Barbalace, Giulia Campanella, Vincenzo Campanella

**Affiliations:** 1 Dental School, Catholic University of Our Lady of Good Counsel, 1000 Tirana, Albania; antlib76@libero.it; 2 Dental School, University of Rome “Tor Vergata”, 00133 Rome, Italy; danilo96m@gmail.com (D.M.); giulia.campanella@students.uniroma2.eu (G.C.); 3 Department of Clinical Sciences and Translational Medicine, Dental School, University of Rome “Tor Vergata”, 00133 Rome, Italy; vincenzo.campanella@uniroma2.it

**Keywords:** core technique, SoftCore, GuttaFusion, bioceramic sealer, gutta-percha, sealer, obturation system

## Abstract

The aim of this preliminary study was to compare two core-carrier obturation techniques—GuttaFusion (GF) and SoftCore (SC)—used in combination with a bioceramic sealer (NeoSealer Flo), and to evaluate their ability to fill simulated root canals. Eight standardized resin models of maxillary first molars were used, and only the P and DV canals of each model were obturated. Cross-sections were obtained at 1 mm and 3 mm from the apex, and the percentage areas occupied by gutta-percha (PGFA), sealer (PSFA), and voids (VA) were measured. This study provides novel comparative data on the performance of these two carrier-based techniques when used with a bioceramic sealer. GF showed higher PGFA and lower PSFA compared with SC at 1 mm from the apex, while SC presented slightly higher VA. At 3 mm, PGFA increased for both techniques. Descriptive statistics (means and percentage values) were calculated; no inferential statistical analysis was performed due to the preliminary nature of the study and the limited sample.

## 1. Introduction

Despite the variety of obturation techniques available, achieving an optimal balance among gutta-percha, sealer, and voids remains a central objective in endodontic therapy [1,2,3]. Carrier-based systems such as SoftCore and GuttaFusion have been introduced to enhance three-dimensional canal filling through the use of thermoplasticized gutta-pecha supported by different core materials. However, although several studies have evaluated the performance of these systems, limited evidence is available on their behavior when used in combination with modern bioceramic sealers [4,5,6]. In particular, the interaction between the carrier material, the viscosity and flow characteristics of the bioceramic sealer, and the resulting distribution of gutta-percha, sealer, and voids has not yet been fully clarified under standardized canal conditions [7,8,9,10].

Based on these considerations, this preliminary study aims to compare the SoftCore and GuttaFusion obturation systems, both used in combination with a bioceramic sealer (NeoSealer Flo), to assess their effectiveness in minimizing excess sealer and optimizing the percentage of gutta-percha filled areas [11,12,13,14,15,16,17]. This preliminary study aims to explore whether the different core materials and thermal properties of SoftCore and GuttaFusion are associated with observable variations in the percentages of gutta-percha, sealer, and void areas at 1 mm and 3 mm from the apex [18]. These apical levels are clinically relevant because proper filling in the final millimeters of the canal is essential to ensure an effective apical seal and reduce the risk of microleakage [19,20,21]

## 2. Materials and Methods

**Figure 1:**
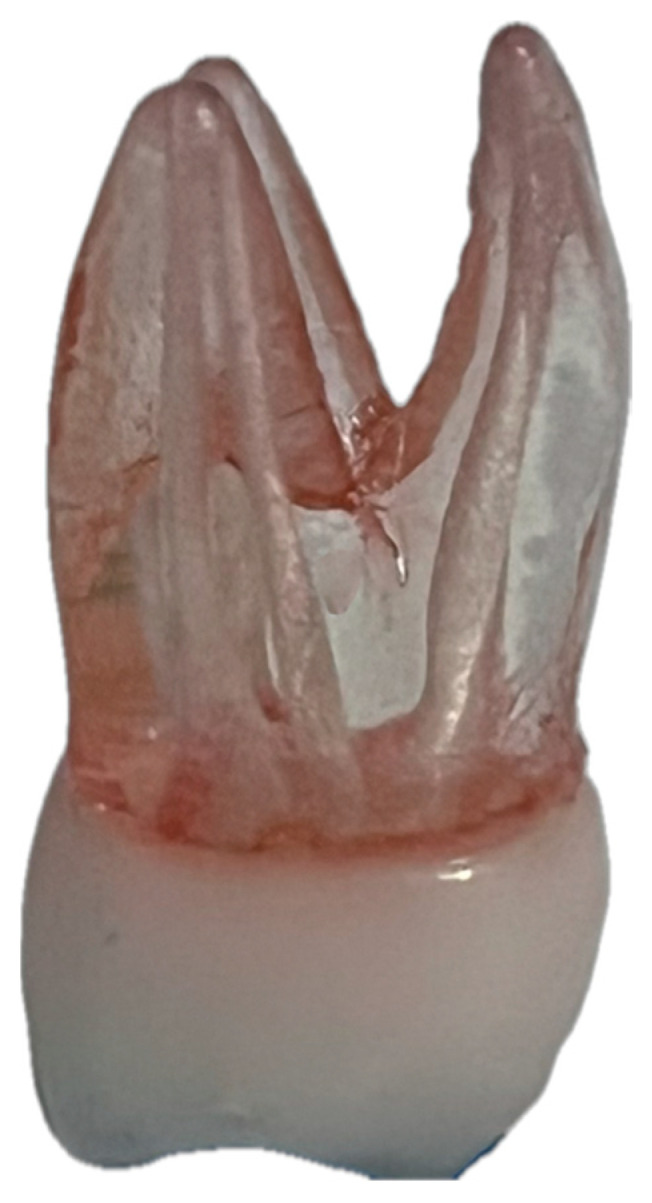
Element model used.

Eight identical resin models of upper first molars were examined (Figure 1). Each model contained simulated canals (canal P, canal DB, canal MB1, and canal MB2). For this study, only the palatal (P) and distobuccal (DB) canals were selected. These canals exhibit more regular, reproducible, and anatomically consistent morphology within the standardized resin maxillary molar models. In contrast, the MB1 and MB2 canals present greater anatomical variability and curvature, which could introduce additional confounding factors. The selection of P and DB canals therefore ensured a higher level of standardization and allowed a more reliable comparison of obturation performance between the two techniques. The canals were instrumented to determine the respective working lengths (WL) for each sample (Table 1).

The working length (WL) was determined using a #10 K-file (Dentsply Maillefer, Bellaigues, Switzerland) until the tip of the instrument became clearly visible at the apical foramen. Mechanical preparation was performed using a single reciprocating file from the “Zarc4Endo” series, the Excalibur 25/05. The file was mounted on a Morita endodontic micromotor set to a reciprocating motion (150°/30°; 450 rpm). During preparation, the canals were irrigated using 30-G side-vented syringes with 2 mL of 5.25% sodium hypochlorite followed by 10 mL of 17% EDTA. Apical gauging was verified using a #30 K-file. All simulated canal samples were then dried with paper points and divided into two groups:

- GuttaFusion (samples 1 to 4);
- SoftCore (samples 5 to 8).

**Figure 2:**
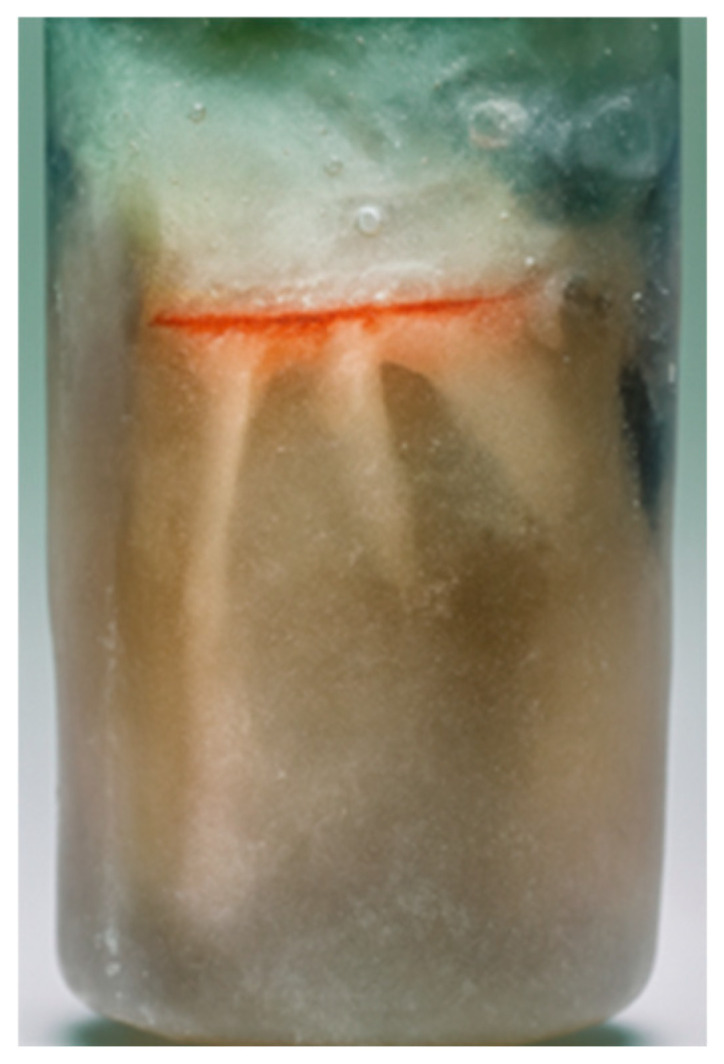
Element model embedded in epoxy resin.

The shaping and filling procedures were performed by a single trained operator following a standardized protocol. A total of 16 CBO 25 obturators (ISO Red color) were selected, with 8 used for the first four samples and the remaining 8 for the last four samples. The obturators were thermoplasticized using the SoftCore Oven (Kerr Endodontics, Orange, CA, USA) [22]. NeoSealer Flo (Avalon Biomed, Houston, TX, USA) was used as the endodontic sealer [23,24,25]. The samples were stored for 14 days at 37 °C and 100% humidity to allow complete setting of the sealer. The teeth were then embedded in epoxy resin blocks (Buehler Ltd., Evanston, IL, USA) (Figure 2).

**Figure 3:**
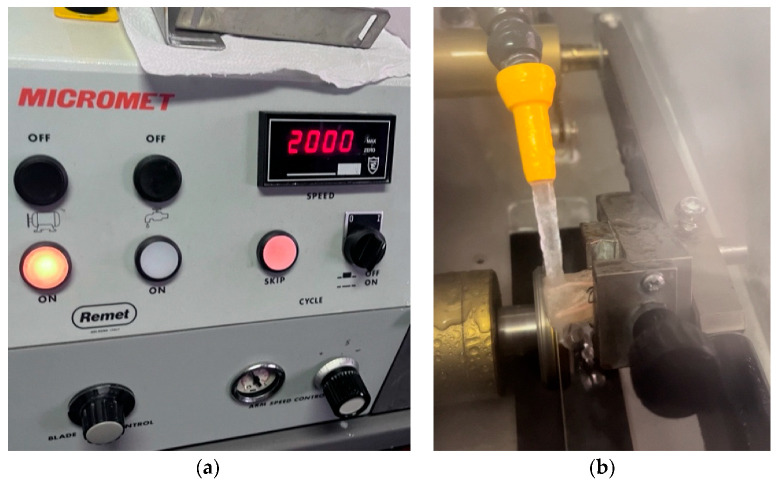
(**a**) The 320 µm cutting machine settings (Remet s.a.s., Bologna, Italy). (**b**) Cutting machine in action.

The teeth were sectioned at 1 mm and 3 mm from the apex, orthogonally to their long axis, using a 320 µm disc cutter (Remet s.a.s., Bologna, Italy) under water cooling (Figure 3a,b). The coronal surface of each section was polished with abrasive papers of progressively finer grit (320, 1200, and 2500) to obtain a smooth surface without noticeable deformation [26]. The sections were then analyzed using a Nikon TS100-F microscope (Nikon, Tokyo, Japan) equipped with a modified light plane at 40× magnification. Images were acquired with an iPhone 15 Pro Max camera (Apple Inc., Cupertino, CA, USA). A standardized protocol was used for the quantitative analysis of the canal cross-sections. All images were exported in TIFF format and analyzed using Adobe Photoshop CS3 (Adobe, San Jose, CA, USA). Before measurement, each image was calibrated by setting the pixel-to-millimeter ratio based on a scale bar obtained under the same magnification. The contours of the total canal area and of each material (gutta-percha, sealer, and voids) were manually traced using the “Lasso Tool” at a fixed 400% zoom level to ensure accuracy and repeatability. For each traced region, the software automatically calculated the pixel count, which was subsequently converted into area values. To minimize operator variability, all measurements were performed twice by the same operator, and the mean value was used for the analysis. Additionally, an estimated percentage representing potential human tracing error was calculated and incorporated into the final reported values. The following areas were evaluated:

- Gutta-percha area;
- Cement area;
- Void area.

The values obtained were expressed as percentages of the total canal area (PGFA, PSFA, and VA). All data were recorded in Excel spreadsheets (Microsoft, Redmond, WA, USA), with corresponding pie charts and histograms.

Additionally, the percentage of error attributable to human tracing during processing in Adobe Photoshop was calculated and incorporated into the final percentage values.

**Figure 4:**
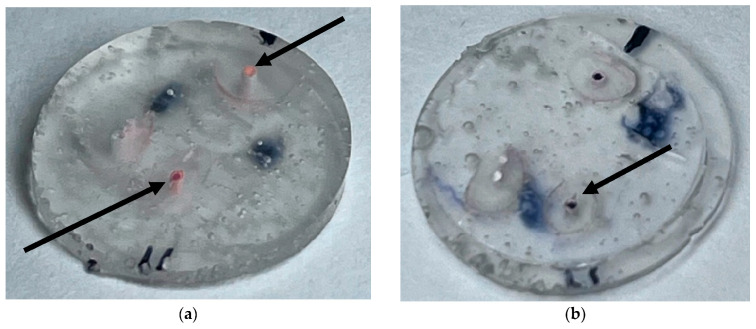
(**a**) Model 4 sectioned 3 mm from the apex (GuttaFusion). (**b**) Model 7 sectioned 3 mm from the apex (SoftCore).

Images of two samples sectioned 3 mm from the apex: the arrows indicate the sections of the DV and P canals (Figure 4a,b)

**Figure 5:**
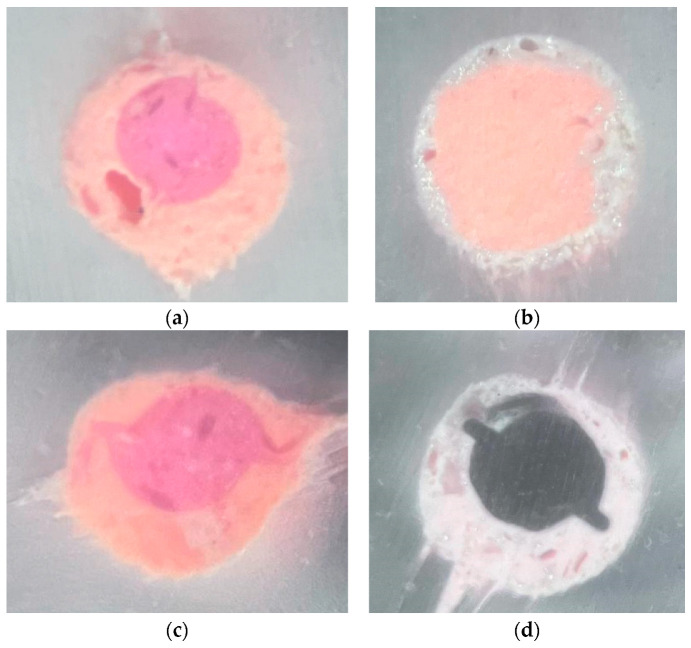
(**a**) Model 4 (GuttaFusion, section 1 mm). (**b**) Model 7 (SoftCore, section 1 mm). (**c**) Model 4 (GuttaFusion, section 3 mm). (**d**) Model 7 (SoftCore, section 3 mm).

Images of the same two samples from Figure 5a–d, with 40× magnification on the palatal canal.

## 3. Results

**Figure 6:**
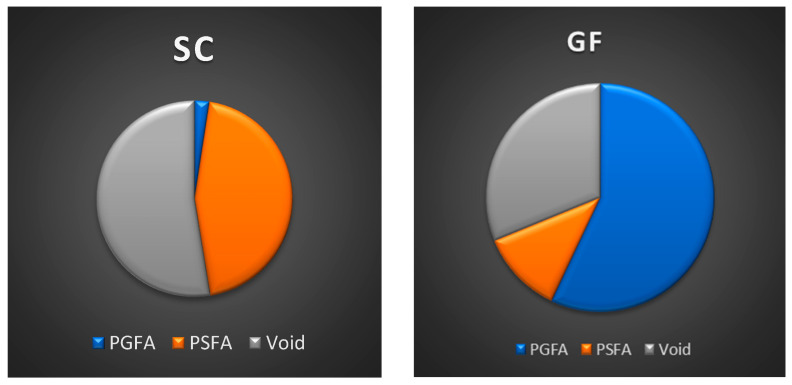
Percentage area of material at 1 mm from the apex palatal root illustrated in pie charts.

**Figure 7:**
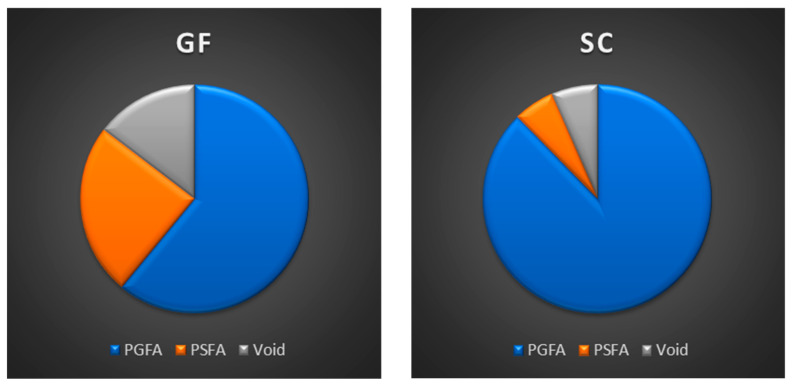
Percentage area of material at 3 mm from the apex palatal root illustrated in pie charts.

When comparing the two techniques in combination with the endodontic sealer, the following results were observed at 1 mm from the apex. GuttaFusion (GF) produced a higher PGFA and a lower PSFA compared with SoftCore (SC) (Figure 6). Additionally, SC showed a higher VA than GF. Similar patterns were observed in the sections obtained at 3 mm from the apex, where PGFA values increased for both techniques (Figure 7). Although no inferential statistical analysis was performed due to the preliminary nature of the study and the limited sample size, the descriptive trends observed may hold clinical interest. In particular, the SC technique demonstrated increased PSFA values compared with GF, with the sealer occupying a substantial portion of the total area. Moreover, SC presented higher VA, whereas GF showed a reduction in voids within the same apical sections.

## 4. Statistical Analysis

All quantitative data for PGFA, PSFA, and VA at 1 mm and 3 mm from the apex were entered into Excel spreadsheets (Microsoft, Redmond, WA, USA) for analysis. For each obturation technique and section level, the percentage values were summarized using descriptive statistics (means and percentage distributions), and the results were illustrated with pie charts and histograms to facilitate visual comparison between groups.

No inferential statistical tests (such as *t*-tests or analysis of variance) were performed, as the study was intentionally designed as a preliminary in vitro investigation with a limited sample size (n = 8 canals in total, 4 per group). Under these conditions, hypothesis testing would not provide reliable estimates of variability or statistical power. Consequently, differences between GuttaFusion (GF) and SoftCore (SC) are interpreted as descriptive trends based on the observed percentage values, rather than as statistically demonstrated effects. References in the Results section to “higher” or “lower” PGFA, PSFA, or VA therefore indicate numerically greater mean percentages within this dataset and should not be understood as statistically significant differences.

## 5. Discussion

The quality of root canal obturation—expressed as the percentage of gutta-percha, sealer, and voids—served as the primary metric for comparing the performance of the two carrier-based systems evaluated in this study [27,28,29]. Achieving a reliable apical seal is essential, particularly considering that only 3–4 mm of canal length typically remain following post-space preparation. Furthermore, the apical third frequently contains lateral canals and anatomical irregularities that may compromise the sealing ability of obturation materials [30]. For this reason, assessments at 1 mm and 3 mm from the apex were selected, as these levels are considered critical for evaluating apical sealing integrity in both clinical and experimental settings. In the present investigation, GuttaFusion (GF) demonstrated a higher percentage of gutta-percha–filled area (PGFA), whereas SoftCore (SC) showed a higher percentage of sealer-filled area (PSFA). Voids (VA) were identified in both groups, with slightly higher values observed in SC, although overall differences remained limited. The contrasting performance between systems can be attributed to the inherent physical and thermal characteristics of their carrier materials. GF employs a cross-linked gutta-percha carrier that facilitates more efficient heat transfer along the obturator, enabling the gutta-percha to achieve a greater degree of plasticization and more intimate adaptation to the canal walls. This mechanism may account for the higher PGFA recorded in the GF samples. Conversely, SC incorporates a polysulfone carrier with reduced thermal conductivity, which may limit the uniform distribution of heat and decrease the flow of thermoplasticized gutta-percha. As a result, a greater amount of sealer may be required to compensate for reduced core adaptability, consistent with the higher PSFA values observed in the SC group. These descriptive trends are consistent with patterns reported in previous laboratory studies evaluating the thermomechanical behavior of core-carrier obturation systems; however, such comparisons should be interpreted cautiously, as the present investigation is exploratory and based on a simplified experimental model. The observations reported here are not intended to suggest clinical equivalence but rather to contextualize the findings within the existing literature [31]. Although the percentage of void areas was similar between techniques, the presence of voids—particularly within the apical 3 mm remains clinically relevant. Voids may compromise the integrity of the apical seal by providing potential pathways for fluid penetration, bacterial ingress, or residual microbial persistence. While the present findings do not reach statistical significance due to the limited sample size, the descriptive trends observed may still hold clinical interest. Importantly, these observations fall within the performance ranges reported in other recent investigations of core-carrier obturation methods [32,33]. Previous literature comparing gutta-percha–based carrier systems, including SoftCore, GuttaFusion, and Thermafil, has generally demonstrated higher gutta-percha-to-sealer ratios relative to warm vertical condensation techniques such as Microseal or System B. In our study, PGFA values at 3 mm were higher than those at 1 mm (in the palatal canals), a trend consistent with prior observations and likely to be related to greater anatomical regularity at slightly more coronal apical levels. Earlier studies also reported that systems such as Thermafil exhibited reduced microleakage compared with lateral condensation or certain warm obturation techniques. Although the PGFA values obtained in this study are slightly lower than those reported for Microseal or System B in comparable studies, they remain within acceptable ranges for carrier-based systems. At the second cutting level, both SC and GF demonstrated reduced VA and increased PSFA, suggesting improved sealer distribution and adaptation. Overall, the values obtained in this preliminary study correspond with previously published evidence and support the characteristic performance of carrier-based obturation systems under standardized experimental conditions.

## 6. Conclusions

Based on the PSFA values, we conclude that in core-carrier endodontic techniques, the use of sealer plays an important—if not essential—role in achieving adequate filling, particularly at 1 mm and 3 mm from the apex. Within the limitations of this preliminary in vitro study, the observed trends may hold potential clinical interest; however, these findings should not be translated into clinical recommendations at this stage. Further studies using natural teeth and larger sample sizes are required to confirm these observations and establish evidence-based guidance for clinical practice.

## 7. Limitations of the Study

This study presents several limitations that must be considered when interpreting the findings. The use of resin-based artificial teeth, while ensuring a high degree of standardization, does not replicate the hardness, dentinal tubule orientation, permeability, or thermal behavior of natural dentin, all of which may influence sealer penetration, heat transfer, and gutta-percha flow. Only two cross-sectional levels (1 mm and 3 mm from the apex) were analyzed, providing a limited representation of the three-dimensional obturation pattern throughout the canal system. The small sample size and the exploratory nature of the design, combined with the absence of randomization, further restrict the generalizability of the results. Additionally, no inferential statistical analyses were performed, and therefore all comparisons should be interpreted as descriptive tendencies rather than evidence of significant differences. Future research using extracted human teeth, a larger number of samples, and more comprehensive imaging modalities would be necessary to validate and expand upon these preliminary observations.

## Figures and Tables

**Table 1:** WL of instrumented canals.

SAMPLES	P ROOT CANAL	DB ROOT CANAL	MB2 ROOT CANAL	MB1 ROOT CANAL
1–8	WL: 22.5 mm Ref: MP cuspid Apex: K-file (30)	WL: 20.5 mm Ref: DV cuspid Apex: K-file (30)	WL: 22 mm Ref: MP cuspid Apex: K-file (30)	WL: 22 mm Ref: MV cuspid Apex: K-file (30)

## Data Availability

The original contributions presented in this study are included in the article. Further inquiries can be directed to the corresponding author.
